# A Case Report of an Osteolytic Mass Hiding Behind an Undiagnosed Parathyroid Adenoma

**DOI:** 10.7759/cureus.74762

**Published:** 2024-11-29

**Authors:** Mohammed S Al Olaimat, Fahad S Al Qooz, Zaid R Alzoubi, Elham M Alsharaiah, Abdulkarim M Alqahtani

**Affiliations:** 1 Maxillofacial Surgery, King Hussein Medical Center, Amman, JOR; 2 Pathology, King Hussein Medical Center, Amman, JOR

**Keywords:** brown tumor, giant cell, hyperparathyroidism, mandible, osteolytic

## Abstract

Hyperparathyroidism is a common endocrinopathy classified into three subtypes: primary, secondary, and tertiary. One of the rare symptoms that patients with hyperparathyroidism present is the formation of osteolytic lesions of the jaws. Brown tumors are rare skeletal osteolytic masses of a poorly understood etiology. These tumors are usually caused by a mutation in the CDC73 gene, a tumor suppressor protein gene that translates to parafibromin. Treatment has been reported to range from a conservative parathyroidectomy to resection and reconstruction. We present a case of a female who presented with a facial disfigurement for four months that was hidden under the effect of a parathyroid adenoma. Our aim is to guide the surgeon in the proper detailed management of osteolytic lesions of jaws that are related to parathyroid hyperfunction.

## Introduction

Brown tumors, also known as "osteitis fibrosa cystica", are rare osteolytic masses accounting for 3% that occur in correlation to an active parathyroid gland. The differential diagnoses affecting the maxillofacial regions are fibrous dysplasia, ossifying fibroma, and giant cell lesions [1,2].

Hyperparathyroidism is a familiar endocrinal disease that is characterized as primary, secondary, and tertiary hyperparathyroidism. The parathyroid gland plays a key role in regulating serum calcium (Ca+2). Parathyroid hormone (PTH) is secreted in response to decreased serum Ca+2. It functions on the outflow of Ca+2 from bony compartments and increases reabsorption by the kidney. Also, PTH leads to increased vitamin D release from the kidneys, which in turn increases the absorption of calcium through the gastrointestinal tract [2].

Brown tumors are lesions that are non-neoplastic in nature with unilocular or multilocular appearances and may affect long bones, pelvis, ribs, and clavicles [3]. The accumulation of hemosiderin pigments is what gives the tumor the so-called “brown tumor,” thus appearing as a friable red-brown mass. Histologically, it is characterized by masses composed of giant cells in a fibrovascular stroma, with cystic-like spaces lined by connective tissue and foci of hemorrhage, also resembling central giant cell granuloma [4-6].

Clinically, patients suffering from hyperparathyroidism may range from being asymptomatic to being severely lethargic. In mild cases, radiographic changes in the jaws may be seen and can give an insight on the patients oral health [6]. Osteoporotic appearances observed in the maxilla and mandible are characterized as "salt and pepper" appearance. In severe cases, they were described as "stones, bones, moans, and groans" which were indicative of renal calculi formation, lower gastrointestinal symptoms, and cognitive issues [6,7].

We present a case of a 49-year-old female that presented with an osteolytic jaw lesion that had no evident clinical signs of hyperparathyroidism. This article has been previously posted in the Authorea preprint server with DOI: https://doi.org/10.22541/au.172114302.27874477/v1.

## Case presentation

**Figure 1:**
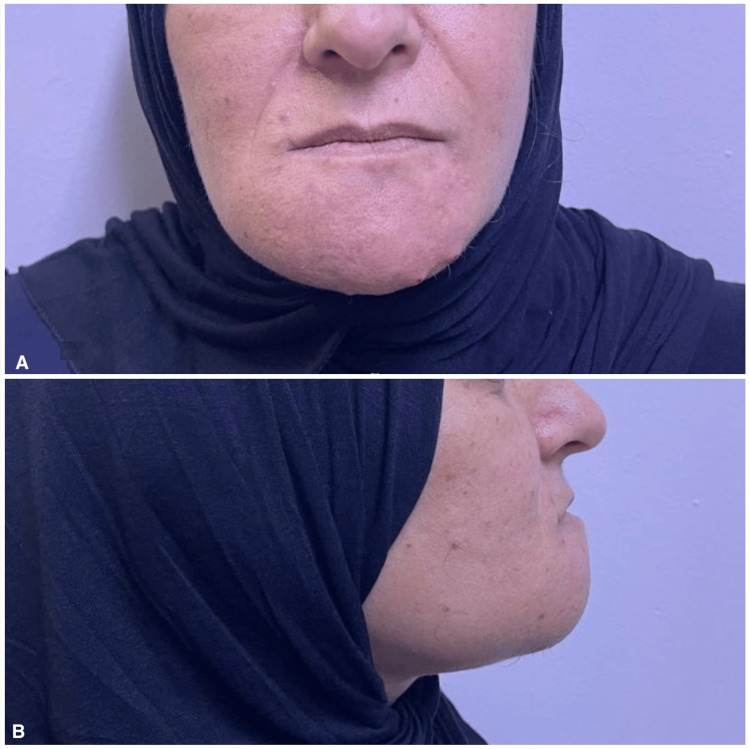
Clinical photograph A. Frontal view showing facial asymmetry and disfigurement due to mass effect. B. Lateral view showing mandibular pseudoprognathism caused by the mass.

**Figure 2:**
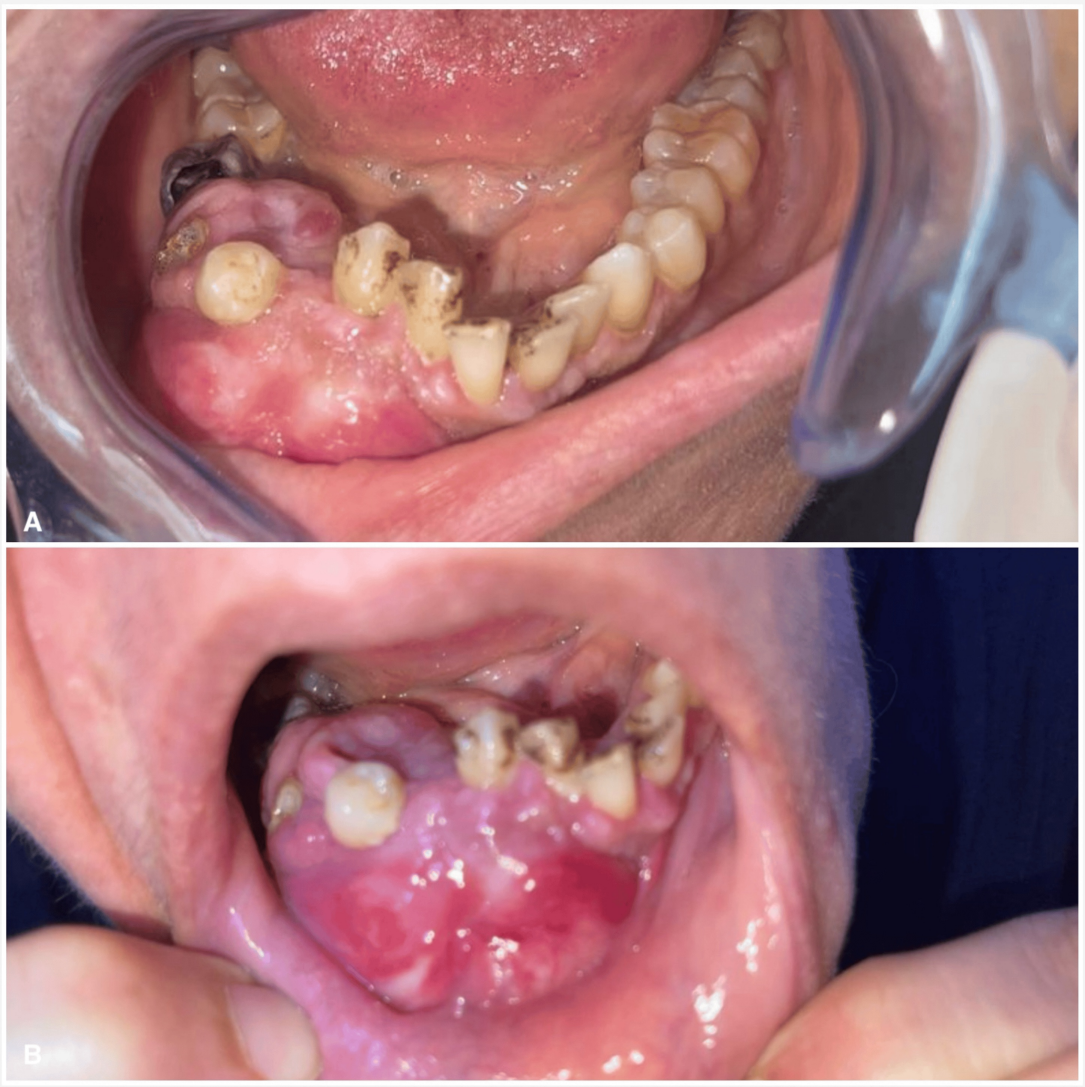
Intraoral photograph A and B showing mandibular buccolingual expansion (brown tumor) on the right side of mandible with displacement of teeth.

A 49-year-old female presented to our Oral and Maxillofacial Surgery clinic at King Hussein Medical Center with expansible right mandibular swelling. She was not known to have any medical problems or allergies. The mass had increased in size within the four months prior to her presentation to our facility. Facial disfigurement and difficulty eating were the patients' main concerns (Figures 1A, 1B, 2A, 2B). The differential diagnoses included ameloblastoma and central giant cell granuloma. Given the image of the mass in the mandible and maxilla, laboratory testing was done. Serum calcium, phosphorus, and parathyroid hormone (PTH) levels were ordered.

Laboratory

Laboratory results reported a PTH level of 876 pg/ml (normal: 15-65 pg/ml), calcium 10.6 mg/dL (normal: 8.4-10.5 mg/dL), and phosphorus 1.52 mg/dL (normal: 2.5-4.5 mg/dL). All other labs were unremarkable. The patient presented back for a follow-up visit, and laboratory results of PTH revealed a level of 1,636 pg/ml, vitamin D levels were within the normal range, and thyroid function tests were unremarkable. The patient was referred to the endocrinology and endocrine surgery departments.

Endocrinology

The endocrinologist saw the patient, and in relation to the patient's laboratory results, their advice was to localize a parathyroid adenoma, administer vitamin D at 50,000 IU weekly, and start on One-Alpha 1 mg one week prior to surgery and, once confirmed by scans, undergo parathyroidectomy.

Radiology

Ultrasound

A neck ultrasound revealed homogeneous thyroid glands with two small cystic nodules in the left lobe of the thyroid gland, the largest measuring 4 mm. A hypoechoic, well-defined nodule is posterior to the middle and lower poles of the right thyroid lobe. The nodule shows significant internal and peripheral vascularity and is likely to represent a right inferior parathyroid adenoma.

 Computed Tomography

**Figure 3:**
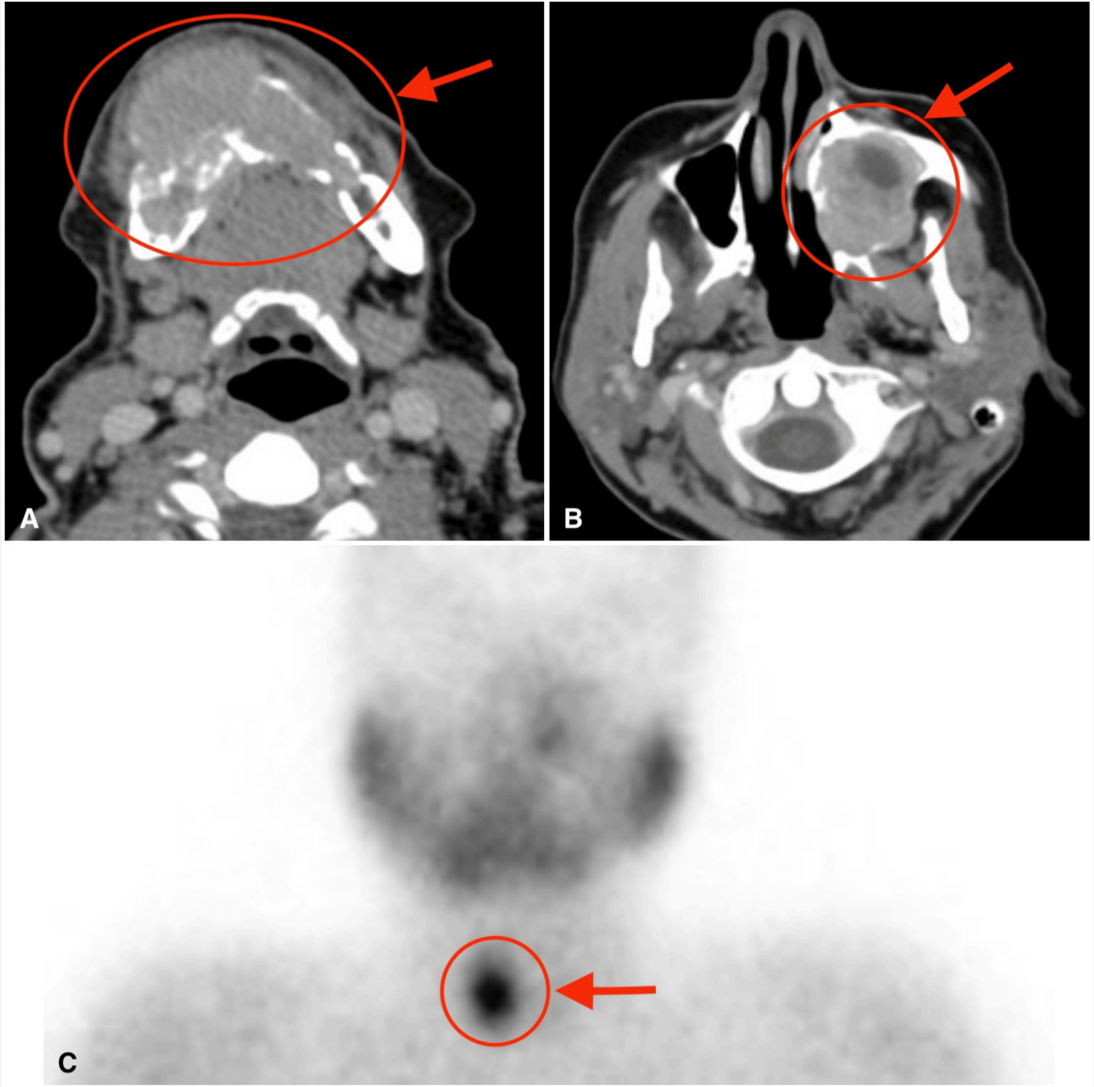
Radiological views A. An axial view (soft-tissue window) CT scan showing an expansile osteolytic multiloculated lesion of the mandibular symphysis and body (red circle and arrow). B. An axial view (soft-tissue window) CT scan shows a left maxillary sinus expansile lesion invading the nasal cavity (red circle and arrow). C. Technetium-99m (Tc-99m) (Sestamibi) shows increased uptake of radiotracer within the right thyroid bed (red circle and arrow).

Upon clinical examination, the mass was found to be located on the right side of the mandible. The mass was nontender and hard, with no signs of an overlying skin infection or previous draining fistulas. There was no neurological deficit. The CT scan reported findings of an expansile lytic lesion with a soap-bubble appearance centered in the right mandibular body, crossing the midline. The mass measured 5 x 3.5 x 1.8 cm in all dimensions (Figure 3A). There was also evidence of a well-defined mass in the left maxilla that was heterogeneous with an expansile nature (Figure 3B). There was also evidence of a hypodense lesion in the right thyroid lobe measuring 10 x 8 mm in full dimensions.

Sestamibi Scan (Technetium-99m)

The scan revealed intense radiotracer retention within the right thyroid bed. As a conclusion with laboratory results, the findings were suggestive of parathyroid functioning adenoma (Figure 3C).

Endocrine surgery

The patient was seen by the endocrine surgeon and was advised to undergo a sestamibi scan. Upon confirmation of the scans, the patient underwent a parathyroid adenectomy, and the specimen was sent for histopathologic examination.

Histopathology

**Figure 4:**
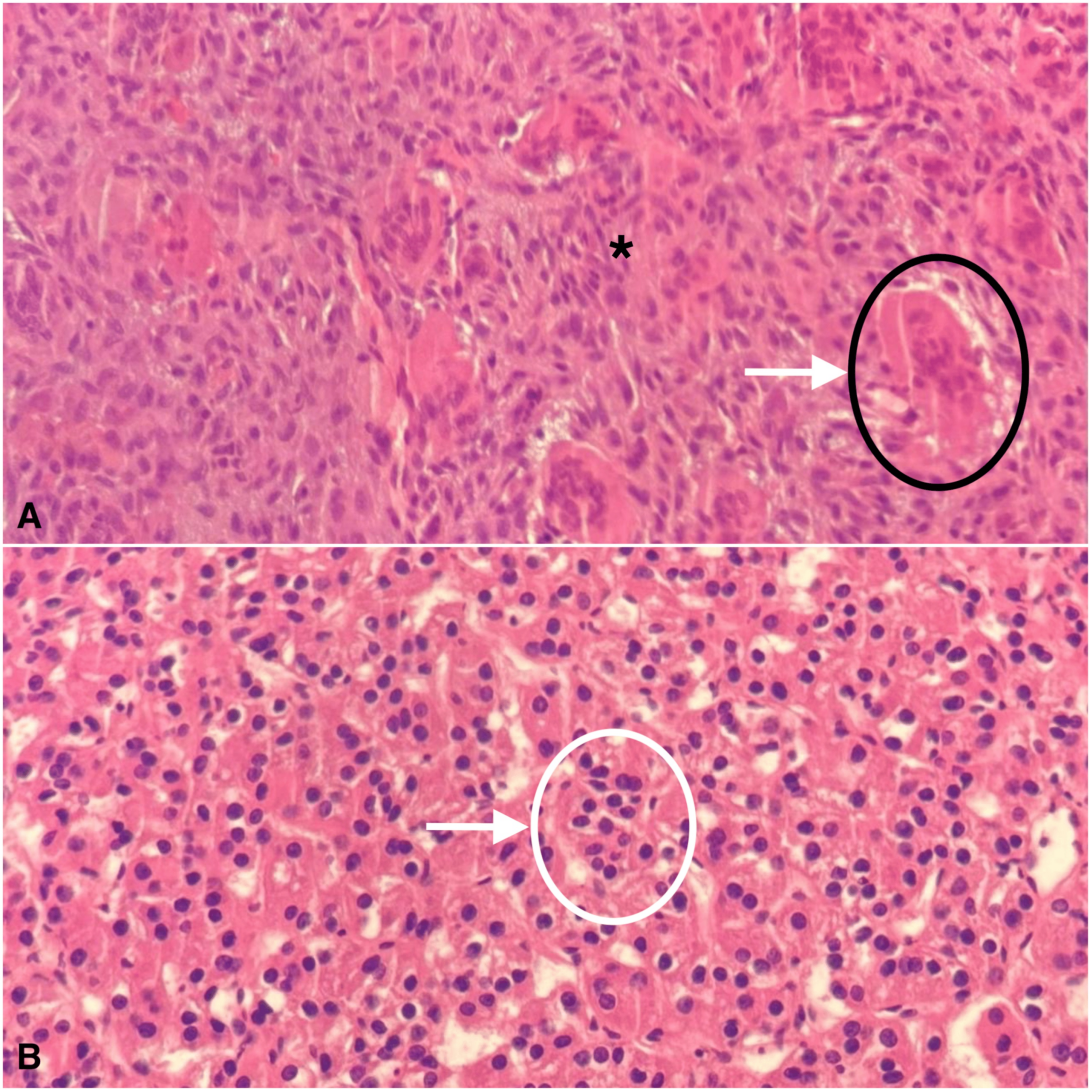
Histopathologic examination A. Mandibular giant cell tumor composed of osteoclast-like giant cells present in a fibrous background stroma (black circle and white arrow) composed of round-oval looking spindle cells (asterisk). B. Parathyroid adenoma: The tumor is composed of chief cells having round nuclei and granular eosinophilic cytoplasm (white circle and arrow).

Multiple sections of fragments reveal a giant cell-rich lesion composed of osteoclast-like giant cells with scattered nuclei and others with an agglomeration of nuclei in the center with a peripheral band of cytoplasm. The cells are present in a background of cellular and vascular stroma composed of mildly pleomorphic, round-oval-looking spindle-shaped cells. Areas of hemorrhage, fibrosis, and osteoid were present without any evidence of necrosis. A histopathologic diagnosis was reported as a giant cell lesion. Differential diagnoses included brown tumor, central giant cell granuloma, and giant cell tumor of bone (Figure 4A). The parathyroid specimen reported the diagnosis of an adenoma (Figure 4B).

## Discussion

Brown tumors were first described by Recklinghausen in 1891 as osteitis fibrosa cystica. This is due to the dysregulation of calcium homeostasis, resulting in the demineralization of bones. Long-standing tumors usually exhibit central degeneration with fibrous stromal replacement. Histologically, the lesions are identical to giant cell tumors that contain spindle cell stroma with multinucleated giant cells. The most common cause of primary parathyroid disease is usually adenoma. It is usually diagnosed by incidental findings of hypercalcemia [2,3,6]. Although our patient did not present with symptoms of hypercalcemia, she demonstrated to us a reasonable disfigurement that was provisionally diagnosed as ameloblastoma. Symptoms of hypercalcemia include nephrolithiasis, muscular weaknesses, osteoporosis, and psychiatric problems [2]. Our patient was medically free and did not suffer from any neurological deficits or side effects of hypercalcemia. Her renal function was within normal range.

The overproduction of PTH results in osteoblastic activity that increases RANKL expression that binds to the RANK receptor on osteoclasts, thus promoting osteoclastic activity. PTH also decreases osteoprotegerin levels, which in turn prevents the RANKL and RANK interactions and inhibits bone resorption [3,6]. Large bimaxillary masses cause facial disfigurements and may compromise daily routine and quality of life [4]. Our patient's concerns were her facial disfigurements and a decreased ability to consume food.

Diagnosis of such masses cannot be done only by histopathologic findings, as they may resemble other bony diseases such as giant cell lesions that include central giant cell granuloma, cherubism, and Langerhans histiocytosis. Therefore, the diagnosis is implicated in clinical, laboratory, radiological, and histopathological findings. The primary goal of such masses is to restore function and management via a surgical resection [4,7].

## Conclusions

Brown tumors frequently occur in hyperparathyroidism, and our patient's presentation of facial disfigurement posed a challenge in diagnosis. Timely and accurate diagnosis is crucial, as it streamlines the process for both the clinician and the patient, preventing unnecessary complications. Laboratory investigations played an essential role in diagnosing this mass in conjunction with the histopathologic examination. In terms of treatment, most patients do not require surgical intervention for the brown tumor itself; the focus should be on addressing the parathyroid adenoma, which often leads to a reduction in mass size over time. Once the parathyroid adenoma is removed, the brown tumor can be managed like any giant cell lesion. Should surgical intervention be necessary, options include debulking, curettage, or resection.
